# Imperatorin ameliorates myocardial ischemia‐reperfusion injury by inhibiting endoplasmic reticulum stress‐mediated apoptosis via the PI3K/AKT/Nrf2 pathway

**DOI:** 10.1002/ccs3.70084

**Published:** 2026-05-22

**Authors:** Mingjun Han, Fan Yu, Huanhuan Li

**Affiliations:** ^1^ Department of Cardiology Hospital of Chengdu University of Traditional Chinese Medicine Chengdu Sichuan China; ^2^ Department of Chronic Non Communicable Disease Prevention and Control Futian Center for Chronic Disease Control Shenzhen China; ^3^ Department of Electrocardiogram Huanggang Central Hospital Huanggang Hubei China

**Keywords:** endoplasmic reticulum stress, imperatorin, myocardial ischemia‐reperfusion injury, PI3K/AKT/Nrf2 pathway

## Abstract

Endoplasmic reticulum stress (ERS) is a major event associated with myocardial ischemia‐reperfusion injury (MIRI). Imperatorin (IMP) has cardioprotective effects, but its role in MIRI has not been reported. H9c2 cells were injured by the hypoxia/reoxygenation (H/R) method. The MIRI mouse model was prepared by coronary artery ligation and by using IMP, and the PI3K/AKT agonist 740 Y‐P and inhibitor LY294002 were used for intervention. Cardiomyocyte apoptosis was determined through flow cytometry and TUNEL staining. Western blot, transmission electron microscopy, calcium assay kits, and Fluo‐4 AM fluorescent probe were used to evaluate ERS and Ca^2+^ levels. In addition, the PI3K/AKT/Nrf2 pathway protein expressions were detected. IMP could significantly reduce cardiomyocyte apoptosis in H9c2 cells and myocardial tissue of MIRI mice, reduce ER swelling and damage, restore ER membrane structure, and reduce Ca^2+^ content and Caspase 12 and C/EBP homologous protein (CHOP) protein levels. IMP also increased p‐PI3K and p‐AKT proteins and the nuclear translocation of Nrf2 in H9c2 cells and myocardial tissue of MIRI mice. After IMP treatment combined with 740 Y‐P intervention, the effect of IMP on improving cardiomyocyte apoptosis and ERS was further promoted, and the apoptosis rate, Ca^2+^ content, and caspase 12 and CHOP protein levels were significantly reduced. LY294002 weakened the improvement of IMP, and apoptosis and ERS levels increased significantly. IMP attenuated MIRI by inhibiting ERS and myocardial apoptosis through activating the PI3K/AKT/Nrf2 pathway.

## Graphical abstract

By activating the PI3K/AKT/Nrf2 pathway, imperatorin can reduce endoplasmic reticulum stress and its mediated apoptosis in cardiomyocytes, improve the pathological changes of myocardial tissue, and thus improve myocardial ischemia/reperfusion injury.

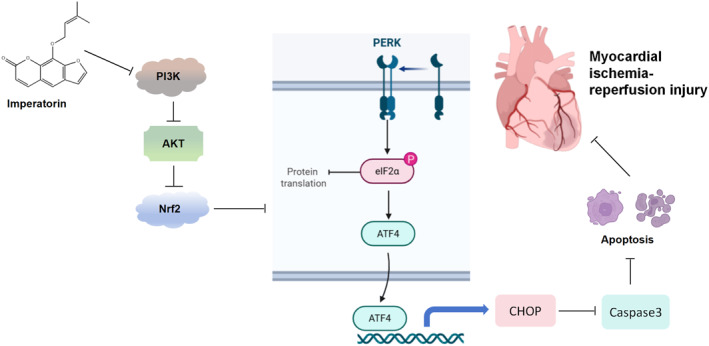

## 1 INTRODUCTION

Acute myocardial infarction (AMI) is a serious heart disease that not only impairs cardiac function, but also significantly reduces patient survival.^1^ Reperfusion therapy is the main effective method for AMI.^2^ Myocardial ischemia/reperfusion injury (MIRI) describes the pathological process where the ischemic heart muscle returns to normal blood flow following a period of partial or complete blockage of the coronary artery and subsequent reopening, yet the damage to the tissue worsens over time.^3^, ^4^ Globally, cardiovascular disease is the leading cause of death, and ischemic heart disease has brought a huge and growing burden to health care systems and societies around the world,^5^, ^6^ which brings heavy economic and medical pressure to families and society as a whole. It is commonly believed that MIRI is an interactive process involving multi‐level and multifactor interactions. It is mainly associated with inflammation, apoptosis, and ferroptosis.^7^, ^8^, ^9^, ^10^, ^11^ The injury mechanisms are interrelated and form an intricate network that jointly promotes MIRI development. Therefore, reducing MIRI is a key method to restore the function of ischemic myocardium. Despite its clinical significance, no specific therapeutic agents are yet available to effectively prevent or treat MIRI in clinical practice.^4^, ^12^ In this context, herbal monomers have the benefits and characteristics of multi‐channel, multi‐target, and multi‐level treatments for MIRI.

Imperatorin (IMP) is a natural furan coumarin that is widely found in various traditional Chinese medicines such as *Angelica dahurica*, *Heracleum hemsleyanum Diels*, and *Angelica sinensis*. It has many pharmacological actions (anti‐tumor, anti‐inflammatory, anti‐oxidation, and cardiovascular protections).^13^ In recent years, IMP has attracted people's attention as a potential heart‐protecting drug. IMP can inhibit lipid accumulation in the aorta through the MAPKs signaling pathway, reduce inflammation, and thus interfere with atherosclerosis.^14^ IMP can reduce cardiac remodeling and left ventricular dysfunction induced by high glucose and high fat by reducing inflammation and oxidative stress.^15^ IMP can reduce obesity, hypertension, and dyslipidemia in rats caused by high fat and high glucose, and improve vascular endothelial injury and vascular morphology.^16^ IMP also has a preventive effect on doxorubicin‐mediated cardiotoxicity by suppressing NLRP3 inflammasome.^17^ These findings suggest that IMP can exert cardioprotective properties through various mechanisms, but it is not clear whether it has an improvement effect on MIRI.

Endoplasmic reticulum (ER) is an important site for protein synthesis, folding, modification, and assembly and is important for maintaining cell homeostasis. ERS‐induced apoptosis is associated with neurodegenerative diseases, atherosclerosis, MIRI, and many other diseases. During myocardial reperfusion, myocardial cells that have lost oxygen and nutrients will undergo calcium metabolism disorders, intracellular oxidative environment damage, and protein folding in the ER lumen. Reduced or misfolding activates ER stress (ERS) protein receptors, resulting in ER homeostasis imbalance, dysfunction, formation of ERS state,^18^ and induction of apoptosis. Recent studies have shown that the excessive stress of ER can be alleviated by degrading unfolded, misfolded proteins, and changes in pro‐apoptotic factors, thereby alleviating myocardial injury.^19^, ^20^ Therefore, appropriate inhibition of ERS during myocardial ischemia‐reperfusion can reduce cardiomyocyte apoptosis, thereby reducing or alleviating MIRI.^21^ So, the modulation of ERS is expected to become an important target for improving MIRI.

Therefore, in order to explore whether IMP can improve MIRI by reducing ERS, this study constructed an MIRI model of H9c2 cardiomyocytes by hypoxia/reoxygenation (H/R) and prepared an MIRI mouse model by coronary artery ligation. The effect of IMP on cardiomyocyte apoptosis and ERS and its related mechanisms were analyzed, and its therapeutic effect was observed, thus providing a theoretical basis for myocardial reperfusion injury treatment and clinical drug application.

## 2 METHODS

### 2.1 Cell culture and modeling

H9c2 cardiomyocytes (RRID: CVCL_0286) were purchased from Shanghai Cell Bank, Chinese Academy of Sciences (SCSP‐5211, Shanghai, China) in October 2023. Upon receipt, the cells were authenticated by the supplier using short tandem repeat (STR) profiling, and no evidence of cross‐contamination or misidentification was found. In order to prevent *Mycoplasma* contamination, the cells were tested using *Mycoplasma* detection kits (CA1080, Solarbio, Beijing, China). All experiments were performed using mycoplasma‐free cells. H9c2 cells were cultured in DMEM complete medium containing 10% fetal bovine serum (G8002, Servicebio, Wuhan, China) and 1% penicillin‐streptomycin (G4015, Servicebio). The cells were maintained at 37°C in a humidified incubator with 5% CO_2_ (WCI‐1200, WIGGENS, Baden‐Württemberg, Germany). Subsequent experiments were performed when cell confluence reached 70%–80%.

H9c2 cells in the logarithmic growth phase were used to construct the MIRI model by the H/R method. H9c2 cardiomyocytes (5 × 10^4^ cells/mL) were seeded in 6‐well plates with 2 mL per well, and 20 μM phosphoinositide 3‐kinase/protein kinase B (PI3K/AKT) agonist 740 Y‐P (S7865, Selleck, Shanghai, China), inhibitor LY294002 (S1737, Beyotime, Shanghai, China), and 0, 10, 20, 40, 80, and 160 μM IMP (T90666, Yuanye, Shanghai, China) were cultured normally for 24 h, and 0 μM IMP was used as a control. After that, the cells were cultured in glucose‐free DMEM medium and placed in a mixed gas (1% O_2_, 94% N_2_, and 5% CO_2_) incubator (New Brunswick, Cambridge, United Kingdom) for 6 h, and then, the cells were replaced with a high‐glucose DMEM medium (with 4500 mg/L glucose). The cells were cultured in a normal incubator at 37°C and 5% CO_2_ for 6 h.

### 2.2 CCK‐8 assay

H9c2 cells were seeded in 96‐well plates. After cell culture and H/R modeling, 10 μL of CCK‐8 solution (G4103, Servicebio) was added under dark conditions and cultured for 2 h. The OD value was measured at a 450‐nm wavelength using a microplate reader (ELX808, BioTek, Beijing, China).

### 2.3 Lactate dehydrogenase (LDH) test to detect myocardial cell injury

After H9c2 cell treatment and H/R modeling, the cell culture medium was collected and centrifuged for 10 min; 100 μL of supernatant was taken, 60 μL of LDH detection solution (KQ105781, Keqiao Biotechnology Co., Ltd., Jiangsu, China) was added to incubate in the dark for 30 min, and the OD value at 490 nm was read by a microplate reader. Each group was tested with three independent replicates, and the relative content of LDH in each group was calculated.

### 2.4 EdU staining

H9c2 cells after H/R were incubated with a 50‐μmol/L EdU reagent (C0071L, Beyotime, Shanghai, China) for 2 h. After removing the culture medium, 1 mL of fixative was added for incubation for 15 min. After washing the cells, 1 mL of permeabilized solution was added for treatment for 15 min. 0.5 mL of reaction solution was added for incubation in the dark for 30 min. After washing the cells, the DAPI reagent was added for nuclear staining, and an anti‐fluorescence quenching mounting agent (G1401, Servicebio) was used for mounting. Laser confocal microscopy (LSM700, Carl Zeiss, Baden‐Württemberg, Germany) was used for observation and photographing. EdU‐positive cells displayed red fluorescence, and the fluorescence intensity positively correlated with the rate of cell proliferation.

### 2.5 Flow cytometry

After the H/R model of H9c2 cells was successfully established, the culture medium on the orifice plate was sucked into the centrifuge tube and marked. 1 mL of trypsin was added to digest the cells. The cells were mixed, and the cell suspension and the washing solution were collected in the corresponding centrifuge tube and centrifuged at 2000 r/min for 3 min. 300 μL of 1× binding buffer was added, and a pipette gun was resuspended and mixed. After being sucked into a 1.5‐mL EP tube, 5 μL annexin V‐APC was added and mixed and incubated for 15 min in the dark. 5 μL PI dye solution was added to the incubate for 5 min in the dark, and 200 μL of 1× binding buffer was added 5 min before the machine was added. Flow cytometry (C6, BD biosciences, New Jersey, United States) was used for detection.

### 2.6 Immunofluorescence

H9c2 cell climbing tablets were prepared. After treatment, the climbing tablets were fixed with 4% paraformaldehyde for 15 min. The goat serum was added dropwise on the climbing film and blocked for 1 h. The primary antibody of C/EBP homologous protein (CHOP, ab11419, abcam) was added dropwise and incubated in a wet box at 4°C overnight. Alexa 488‐labeled fluorescent secondary antibody (GB25303, Servicebio) was added dropwise to the slides and incubated in a wet box for 1 h. DAPI was added and incubated for 5 min in the dark. The specimens were stained with nuclei, and PBST was used to wash away the excess DAPI. The relative fluorescence intensity of CHOP in H9c2 cardiomyocytes was observed under a fluorescence microscope (Odyssey, Laikuo Biotechnology Co., Ltd., Beijing, China).

### 2.7 Fluo‐4 AM fluorescent probe

H9c2 cells were cultured in a special confocal culture dish. After the cell intervention, a Fluo‐4 AM fluorescence kit (MA0196, Meilun Biotechnology Co., Ltd., Dalian, China) was used for Ca^2+^ fluorescence probe staining. Firstly, the Fluo‐4 AM mother liquor was incubated in water at 25°C for a moment until it was completely melted, and then, it was diluted to a final concentration of 2 mmol/L Fluo‐4 AM working solution using HBSS solution. Then, the diluted Fluo‐4 AM working solution was added to H9c2 cells and cultured at 37°C and 5% CO_2_ for 20 min. Subsequently, 2.5 mL of HBSS solution containing 1% fetal bovine serum was added and continued to be cultured for 40 min. The cells were washed three times with HEPES buffer (10 mmol/L HEPES, 1 mmol/L Na_2_HPO_4_, 137 mmol/L NaCl, 5 mmol/L KCl, 1 mmol/L CaCl_2_, 0.5 mmol/L MgCl_2_, and 5 mmol/L glucose, 0.1% BSA, pH7.4), and then, the cells were resuspended with HEPES buffer to prepare a solution of 1 × 10^5^ cells/mL. After incubation at 37°C for 10 min, fluorescence microscopy was used to observe and collect images.

### 2.8 Animal grouping and drug intervention

Thirty 8‐week‐old healthy SPF C57BL/6J male mice, weighing 20–25 g, were purchased from Spyford Biotechnology Co., Ltd. (Beijing, China). The mice were fed with feed and pure drinking water for 1 week at room temperature 22–24°C, relative humidity 50%–60%, and 12 h/12 h alternating light and dark in an SPF clean animal room. The experimental protocol has been approved by the Hospital of Chengdu University of Traditional Chinese Medicine Ethic Committee (Approval No: KY‐‐202401032; Date: 2024.1.3).

The mice were randomly divided into 5 groups according to the random number table method, with 6 mice in each group: sham operation (Sham) group, model (MIRI) group, MIRI + IMP treatment group, MIRI + IMP+740Y‐P group, and MIRI + IMP + LY294002 group. The mice in the IMP group were intraperitoneally injected with 25 mg/kg IMP (dissolved in normal saline containing 1% DMSO); the mice in the IMP+740Y‐P group and the IMP + LY294002 group were intraperitoneally injected with 20 mg/kg 740Y‐P and 30 mg/kg LY294002, respectively, at the same time of intraperitoneal injection of 25 mg/kg IMP. The MIRI model was established by coronary artery ligation 3 h later.^22^ The mice were anesthetized by intraperitoneal injection of 50 mg/kg pentobarbital sodium and placed on the operating table in a lateral position. After tracheal intubation and mechanical ventilation of the small animal ventilator, the skin was obliquely cut 1–2 cm along the third and fourth intercostals of the left margin of the sternum. The local muscle tissue was separated layer by layer to expose the heart. The surgical field was expanded with a breast expander, and the pericardium was torn open. An 8‐0 surgical suture was used to pass through the left anterior descending coronary artery (LAD) at about 1 cm below the left atrial appendage and the arterial cone, and the blood vessels were ligated. It was observed that the local myocardial tissue of the left ventricular anterior wall and the ligation site became grayish white, indicating successful myocardial ischemia. Then, the chest was simply closed. After 30 min of ischemia, the ligature was removed using ophthalmic scissors, allowing reperfusion for 90 min to restore blood flow. At this time, the myocardial tissue of the mice gradually recovered from gray to light red. The chest cavity was cleaned, the chest wall muscles and skin were sutured layer by layer, and the mice were placed on an electric blanket to rewarm. The mice in the Sham group only underwent the same operation but did not ligate the LAD. After 24 h of MIRI, the mice were fully anesthetized, and the hearts of the mice were taken and weighed.

### 2.9 Western blot

After the H/R model of H9c2 cells was completed, the cell culture medium was removed, PBS was washed twice, 100 μL RIPA lysate (G2002, Servicebio) was added, and the ice was lysed for 30 min. After centrifugation, the supernatant was collected. The myocardial tissue in the ischemic area of mice was cut into small pieces and placed in a homogenizer tube. Homogenizer beads and RIPA lysate were added. The ice was lysed for 30 min, and the supernatant was collected by centrifugation. The BCA protein concentration assay kit (G2026, Servicebio) was used to determine the protein concentration of the samples. After the sample protein was separated by SDS‐PAGE electrophoresis, the protein was transferred to the PVDF membrane, blocked with 5% skimmed milk powder for 2 h, and diluted primary antibody was added and then incubated overnight at 4°C. The TBST membrane was washed three times, and IgG‐HRP labeled secondary antibody (ab6734, 1:5000, abcam) was added to incubate for 60 min. The PVDF membrane was placed on a chemiluminescence instrument, and the target band was developed and exposed by the ECL chemiluminescence method. The gray value of each group of bands was analyzed by Image J software.

Following were the primary antibodies used in this study: cysteinyl aspartate specific proteinase 3 (Caspase 3, ab184787, 1:2000, Abcam), cleaved caspase‐3 (ab214430, 1:5000, Abcam), B‐cell lymphoma‐2 (BCL‐2, ab194583, 1:2000, Abcam), protein kinase R‐like ER kinase (PERK, ab229912, 1:1000, Abcam), p‐PERK Thr980 (3179, 1:1000, cell signaling, Danvers, MA, USA), inositol‐requiring enzyme 1α (IRE1α, 3294, 1:1000, cell signaling), glucose‐regulated protein 78 (GRP78, ab108613, 1:1000, Abcam), activating transcription factor 4 (ATF4, ab216839, 1:1000, Abcam), CHOP (ab11419, 1:1000, Abcam), phosphoinositide‐3 kinase (PI3K, 4292, 1:1000, cell signaling), p‐PI3K Tyr458 (17366, 1:1000, cell signaling), BCL‐2–associated X protein (BAX, ab182733, 1:2000, Abcam), protein kinase B (AKT, ab314110, 1:1000, Abcam), p‐AKT Ser473 (ab314038, 1:5000, Abcam), nuclear factor E2 related factor 2 (Nrf2, ab313825, 1:3000, Abcam), caspase 12 (ab62484, 1:2000, abcam), Lamin B1 (ab229025, 1:1000, Abcam), and GAPDH (ab181603, 1:10000, Abcam).

### 2.10 Real‐time quantitative polymerase chain reaction

Total RNA was extracted from cells using TransZol Up reagent (ET111‐01‐V2, TRANS, Beijing, China). After RNA concentration was detected, RNA was reverse‐transcribed into cDNA according to the instructions of the reverse transcription kit (2621, TAKARA, Tokyo, Japan). According to the TB Green FAST qPCR kit (CN830S, TAKARA) instructions, 2 μL cDNA was used as a template, and the system was mixed. The gene fragment was amplified by real‐time PCR using β‐actin as an internal reference. Reaction conditions were as follows: pre‐denaturation at 95°C for 30 s; denaturation at 95°C for 5 s, annealing at 60°C for 10 s, a total of 40 cycles. The data were analyzed by 7300 System SDS software, the 2^−ΔΔCt^ values of each group were calculated, and the expression levels of RNA in each group were compared. The primer sequences of NOQ1, HO‐1, and β‐actin were as follows: NADPH/quinone oxidoreductase 1 (NOQ1): F: 5′‐GACATCACAGGGGAGCCG‐3'; R: 5′‐CTCAGGCGGCCTTCCTTATAC‐3'; heme oxygenase‐1 (HO‐1): F: 5′‐GTGCACATCCGTGCAGAGAA‐3'; R: 5′‐GTGCACATCCGTGCAGAGAA‐3'; β‐actin: F: 5′‐CACGATGGAGGGGCCGGACTCATC‐3'; and R: 5′‐TAAAGACCTCTATGCCAACACAGT‐3'.

### 2.11 TTC staining

The mouse heart was quickly isolated and placed in a refrigerator at −20°C for 30 min. The heart was taken out, and 5–6 slices were made, with a thickness of about 2 mm. The sections were placed in 2% triphenyltetrazolium chloride (TTC) solution (G1017, Servicebio) in a dark water bath at 37°C for 30 min, and the container was slightly shaken every 5 min to stain the solution fully. Photos were taken under a microscope (NIKON ECLIPSE E100, Nikon, Tokyo, Japan). The white area was the myocardial infarction area, and red area was the myocardial noninfarcted area. Using Image J software, the area at risk (AAR) and infarct area (IA) were measured, and the infarct size was expressed as IA/AAR (%).^23^, ^24^

### 2.12 Masson staining

The fresh myocardial tissue in the ischemic area was fixed in 4% paraformaldehyde, dehydrated, transparent, paraffin‐embedded, and cut into 5‐μm thick slices. The heart sections covered with paraffin were dewaxed in water, soaked in potassium dichromate, and allowed to stand overnight. Then, the sections were stained in iron hematoxylin staining solution for 1 min and rinsed with running water. The samples were dyed with Ponceau magenta and aniline blue, washed with glacial acetic acid, dehydrated with ethanol, and then sealed with neutral gum. Images were collected under a microscope, and the area of myocardial fibrosis was analyzed using Image J software.

### 2.13 TUNEL staining

The sections of the heart covered with paraffin were dewaxed by gradient ethanol, and the heart tissue was covered with protease K working solution. The heart tissue was incubated in a 37°C incubator for 30 min. Then, 0.5% Triton X‐100 was added dropwise, incubated for 20 min, and washed. The TUNEL reaction solution was prepared following the kit instructions (G1504, Servicebio) and added dropwise to the sections. The sections were incubated at 37°C for 1 h and stained with DAPI dropwise. After incubation in the dark for 5 min, the tablets were sealed with an anti‐fluorescence quenching sealing agent. Five nonoverlapping high‐power fields of view in the slice were randomly selected, and the images were observed and collected using a fluorescence microscope.

### 2.14 Transmission electron microscope

The H9c2 cells in each group were digested and centrifuged at 3000 r/min at 4°C. The supernatant was discarded, fixed with 2.5% glutaraldehyde for 24 h, and then fixed with PBS and 1% acetic acid for 2 h. After dehydration and soaking, they were baked at 40°C and 60°C for 12 and 48 h, respectively, and sliced to a thickness of 70 nm. At the same time, 1 mm^3^ of fresh myocardial tissue in the ischemic area was quickly taken and fixed in the electron microscope fixative at 4°C in the dark for 3 h. After rinsing, the samples were fixed with 1% osmic acid, dehydrated, embedded in epoxy resin, and cut into ultra‐thin sections (70 nm in thickness). Then, the cells and tissue sections were stained with uranyl acetate–lead citrate, the ultrastructure of ER was observed by transmission electron microscopy (HT7800, HITACHI, Tokyo, Japan), and the images were collected.

### 2.15 Kit determination

The content of Ca^2+^ in H9c2 cells and mouse myocardial tissue was detected according to the Ca^2+^ kit (C004‐2‐1, Jiancheng Biological Research Institute, Nanjing, China) instruction. The calcium ion in the sample was combined with methyl thymol blue (MTB) in an alkaline solution to form a blue complex. The content of calcium in the sample can be calculated by comparing the color with the calcium standard of the same treatment. H9c2 cells were digested with trypsin, and the cell precipitate was collected after centrifugation. The cells were suspended in 0.5 mL of deionized water, ultrasonically disrupted, and centrifuged for 10 min. The mouse myocardial tissue was added with 9 times the volume of deionized water and homogenized on ice. After centrifugation for 10 min, 10% homogenate supernatant was taken for testing. Three regions in the 96‐well plate were designated as control holes, standard holes, and sample holes. The reagents were added to each hole according to the instructions and then incubated for 5 min. The OD value was measured at a 610‐nm wavelength using a microplate reader.

After anesthesia, the eyeballs of the mice were removed for blood collection, and the supernatant was taken by centrifugation. In strict accordance with the instructions of creatine kinase‐MB (CK‐MB, SEKM‐0152, Solarbio) and LDH (BC0685, Solarbio) content detection kits, the absorbance values were measured using a microplate reader (680, Bio‐Rad, California, United States); CK‐MB and LDH contents were calculated through formulas and standard curves, respectively.

### 2.16 Statistical analysis

Statistical analysis was performed using SPSS 27.0. The experiments were performed with at least 3 independent biological replicates and in 3 technical replicates each. All data were tested for normality and homogeneity of variance. The Shapiro–Wilk test was used to confirm the normality of the data, and the Levene test was used to test variance consistency. If the data were normal and uniform in variance, one‐way ANOVA was used for comparison between multiple groups, and then, Tukey's honest significant difference test was performed as a post hoc analysis. If the data did not meet the variance normality or homogeneity assumption, the Kruskal–Wallis test was used for comparison between multiple groups, and Dunn's test was used for post hoc analysis. The data of each group were expressed as mean ± standard deviation. *p* < 0.05 was considered statistically significant.

## 3 RESULTS

### 3.1 IMP ameliorated H/R‐induced decline in cardiomyocyte viability and apoptosis

**FIGURE 1:**
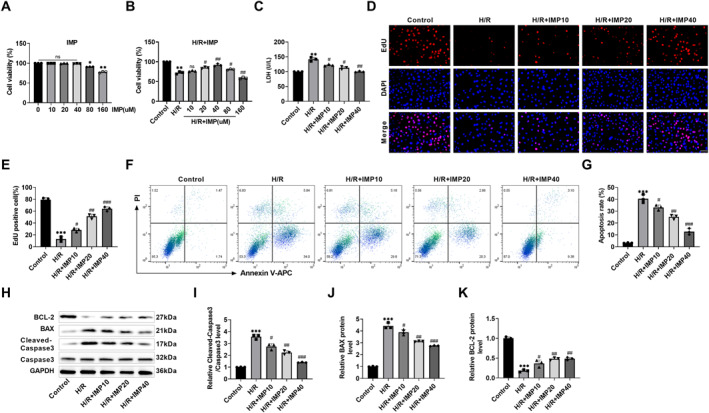
IMP ameliorated H/R‐induced decrease in cardiomyocyte viability and apoptosis. (A) After different concentrations of IMP‐trained H9c2 cells, the cell viability was detected by the CCK‐8 kit. IMP at 40 μM and below did not have any influence on cell viability. (B) IMP treated H9c2 cells for 24 h. The myocardial ischemia/reperfusion injury model was constructed through the H/R method, and the cell viabilities were determined with the CCK‐8 kit. IMP at 40 μM and below increased cell viability. (C) The H9c2 cytotoxicity was detected by LDH kits. IMP reduced LDH content. (D and E) Multiplication of H9c2 cells was detected by EdU staining. IMP increased the number of EdU positive cells (×40, 50 μm). (F and G) The cell apoptosis level was determined by flow cytometry. IMP reduced apoptosis. (H–K) Apoptosis‐related protein expressions were detected by western blot. After IMP treatment, cleaved caspase 3 and BAX levels were lessened, and the BCL‐2 level was raised. *n* = 3, *p < 0.05, **p < 0.01, and ***p < 0.001 versus control group; #*p* < 0.05, ##*p* < 0.01, and ###*p* < 0.001 versus the H/R group. Due to the limited sample size, statistical comparisons involving more than two groups should be interpreted with caution; the reported *p*‐values were considered exploratory. IMP, imperatorin; LDH, lactate dehydrogenase.

This research investigated IMP effects on H/R‐elicited H9c2 cardiomyocytes. We used 0–160 μM IMP to intervene in H9c2 cells for 24 h. There were no remarkable differences in H9c2 cell viability when IMP concentration was ≤40 μM. However, when the concentrations of IMP were 80 and 160 μM, H9c2 cell viability was reduced (Figure 1A), suggesting that low concentrations of IMP had no toxic damage effect on H9c2 cells. After H9c2 cardiomyocytes were treated with IMP, the MIRI model of H9c2 cells was constructed by the H/R method. IMP at 40 μM and below increased the viability of H9c2 cells in the H/R model, and 40 μM IMP had the best intervention effect. IMP at 80 and 160 μM significantly reduced cell viability (Figure 1B). Based on this, 10, 20, and 40 μM IMP were selected for subsequent experiments. LDH is an important indicator of cell damage. LDH assay revealed that H/R caused an increase in LDH content, indicating that H/R caused damage in H9c2 cardiomyocytes. However, IMP treatment showed a trend toward reducing LDH content (Figure 1C), indicating that IMP reduced the damage of H9c2 cells. Secondly, EdU positive cells were decreased after H/R and increased after H/R + IMP treatment (Figure 1D, E), indicating that H/R + IMP promoted the growth of H9c2 cells. Conversely, the apoptosis rate increased after H/R and decreased after IMP treatment (Figure 1F, G), indicating that IMP inhibited H9c2 cell apoptosis. Finally, after H/R induction, cleaved caspase 3 and BAX protein expressions raised, and BCL‐2 protein level decreased. IMP treatment showed a trend toward reversing the levels of apoptotic proteins mentioned above (Figure 1H–K). The above experiments showed that IMP inhibited cardiomyocyte apoptosis and promoted cell proliferation.

### 3.2 IMP improves H/R‐induced ERS in cardiomyocytes

**FIGURE 2:**
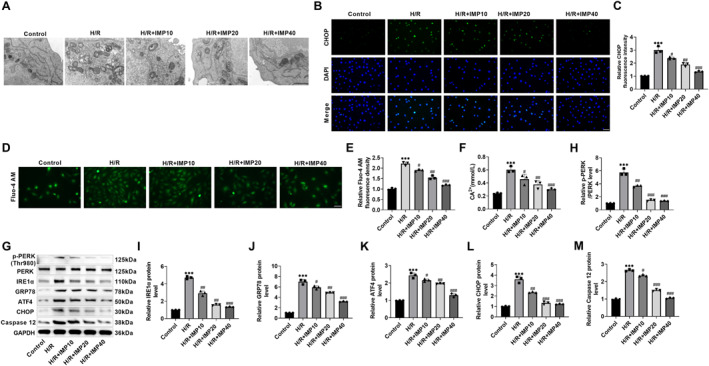
IMP improves H/R‐elicited ERS in cardiomyocytes. (A) ER morphology in each group was observed by transmission electron microscopy. H/R can cause ER swelling and damage, ER cavity expansion, and omentum structure damage; IMP improved this ER damage (×15.0k, 1 μm). (B and C) ERS marker CHOP level was examined through immunofluorescence. The fluorescence intensity of CHOP increased after H/R induction and decreased after IMP intervention (×40, 50 μm). (D and E) Intracellular Ca^2+^ concentration was detected by the Fluo‐4 AM fluorescent probe, which increased after H/R induction and decreased after IMP intervention (×40, 50 μm). (F) The intracellular Ca^2+^ content was determined with a calcium assay kit. The content of Ca^2+^ increased after H/R induction and decreased after IMP intervention. (G–L) Western blot detection of change trend in ERS regulatory proteins. After IMP treatment, the expression of p‐PERK, IRE1α, glucose‐regulated protein 78, activating transcription factor 4, and CHOP proteins was reduced. *n* = 3, ***p < 0.001 versus control group; #*p* < 0.05, ##*p* < 0.01, and ###*p* < 0.001 versus H/R group. Due to the limited sample size, statistical comparisons involving more than two groups should be interpreted with caution; the reported *p*‐values were considered exploratory. CHOP, C/EBP homologous protein; ER, endoplasmic reticulum; ERS, endoplasmic reticulum stress; IMP, imperatorin.

Ischemia‐reperfusion injury can cause ERS, activate ER‐related proteins such as CHOP, and ultimately cause irreversible damage to myocardial tissue. Using transmission electron microscopes (TEMs), it was found that the ER of H9c2 cells was damaged after H/R induction, which was manifested by the swelling and damage of the ER, the expansion of ER cavity, and the destruction of omentum structure, suggesting that H/R induced ERS. However, after IMP treatment, the ER damage was improved, the swelling was alleviated, and the omentum structure was restored (Figure 2A). CHOP is a recognized marker of ERS. The fluorescence intensity of CHOP in H9c2 cells increased after H/R induction and decreased after IMP intervention (Figure 2B, C), indicating that IMP improved the ERS of H9c2 cells. ERS can cause Ca^2+^ balance disorder, so the increase of cytoplasmic Ca^2+^ concentration is a common mechanism of abnormal ERS. The Fluo‐4 AM fluorescent probe was used for the detection of intracellular Ca^2+^ concentration. The green fluorescence intensity increased after H/R induction. After administration of IMP, the green fluorescence intensity in the cells decreased to varying degrees (Figure 2D, E), indicating that IMP showed a trend toward reducing the increase of Ca^2+^ concentration in the H9c2 cytoplasm induced by H/R. At the same time, the calcium assay kit also detected that the concentration of Ca^2+^ in H9c2 cells increased after H/R induction and decreased after IMP treatment (Figure 2F). In order to explore the trend of ERS regulatory proteins, we found that the levels of p‐PERK, IRE1α, GRP78, ATF4, and CHOP increased after H/R induction and decreased after IMP treatment by western blot (Figure 2G–L). We examined the protein expression level of ER stress‐specific initiation caspase. H/R stimulation upregulated the expression of caspase 12, while IMP intervention reversed this change in a dose‐dependent manner (Figure 2M). This suggested that the anti‐apoptotic effect of IMP was at least partly due to its inhibition of ER stress‐specific apoptotic pathways. Therefore, IMP may improve H/R‐induced ERS by regulating cytoplasmic Ca^2+^ homeostasis and ERS regulatory protein expression in cardiomyocytes, thereby improving cardiomyocyte apoptosis.

### 3.3 IMP activated the PI3K/AKT/Nrf2 pathway

**FIGURE 3:**
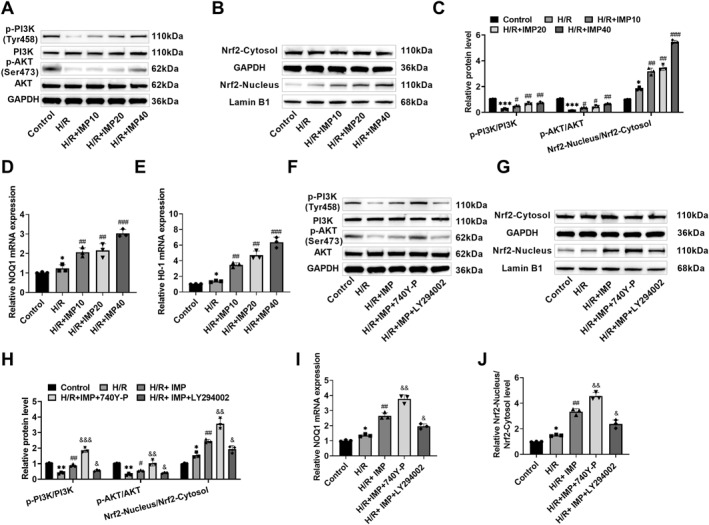
IMP activated the PI3K/AKT/Nrf2 pathway. (A–C) The change trend of the PI3K/AKT/Nrf2 signaling pathway protein was determined through western blot. IMP treatment increased p‐PI3K, p‐AKT, and Nrf2 nucleus expressions. (D and E) The expression levels of downstream target genes NQO1 and HO‐1 in the Nrf2 signaling pathway were detected by RT‐qPCR. IMP treatment increased NQO1 and HO‐1 RNA expression levels. (F–H) H9c2 cells were treated with the PI3K/AKT agonist 740 Y‐P and inhibitor LY294002 at the same time as IMP intervention, and the change trend of the PI3K/AKT/Nrf2 pathway protein was determined through western blot. The protein levels of p‐PI3K, p‐AKT, and Nrf2 nucleus increased after 740 Y‐P intervention and decreased after LY294002 treatment. (I and J) The expression levels of NQO1 and HO‐1 were detected by RT‐qPCR. The expression levels of NQO1 and HO‐1 RNA increased after 740 Y‐P treatment and decreased after LY294002 treatment. *n* = 3, *p < 0.05, **p < 0.01, and ***p < 0.001 versus control group; #*p* < 0.05, ##*p* < 0.01, and ###*p* < 0.001 versus H/R group; &*p* < 0.05, &&*p* < 0.01, and &&&*p* < 0.001 versus H/R + IMP group. Due to the limited sample size, statistical comparisons involving more than two groups should be interpreted with caution; the reported *p*‐values were considered exploratory. IMP, imperatorin; RT‐qPCR, real‐time quantitative polymerase chain reaction.

The PI3K/AKT pathway mediates Nrf2 activation and nuclear translocation. After H/R induction, p‐PI3K and p‐AKT expressions were obviously lessened, Nrf2 cytosol expression did not change, and Nrf2 nucleus protein levels increased slightly; however, after IMP treatment, p‐PI3K, p‐AKT, and Nrf2 nucleus expressions were raised (Figure 3A–C), suggesting that IMP could increase H/R‐induced downregulation of p‐PI3K and p‐AKT expressions and nuclear translocation of Nrf2. Moreover, NQO1 and HO‐1 are downstream target genes of the Nrf2 signaling pathway. Real‐time quantitative polymerase chain reaction (RT‐qPCR) results showed that the expression levels of NOQ1, and HO‐1 RNA were increased after IMP treatment (Figure 3D, E). Since in the previous study, 40 μM IMP had the best improvement effect, this concentration was selected for subsequent experiments. In this study, 740 Y‐P and LY294002 were used to interfere with the pathway PI3K/AKT in H9c2 cells. The results showed that compared with IMP intervention, 740 Y‐P treatment increased p‐PI3K, p‐AKT, and Nrf2 nucleus expressions and NOQ1 and HO‐1 RNA expression levels, while LY294002 intervention reduced p‐PI3K, p‐AKT, and Nrf2 nucleus protein expressions and NOQ1 and HO‐1 RNA expression levels (Figure 3F–J). This again shows that IMP activated the PI3K/AKT signaling to promote the nuclear translocation of Nrf2. Therefore, this study suggested that IMP had a protective impact against H/R‐induced cardiomyocyte damage, and its mechanism was related to the activation of the PI3K/AKT/Nrf2 pathway.

### 3.4 IMP ameliorated H/R‐induced cardiomyocyte apoptosis by the PI3K/AKT/Nrf2 pathway

**FIGURE 4:**
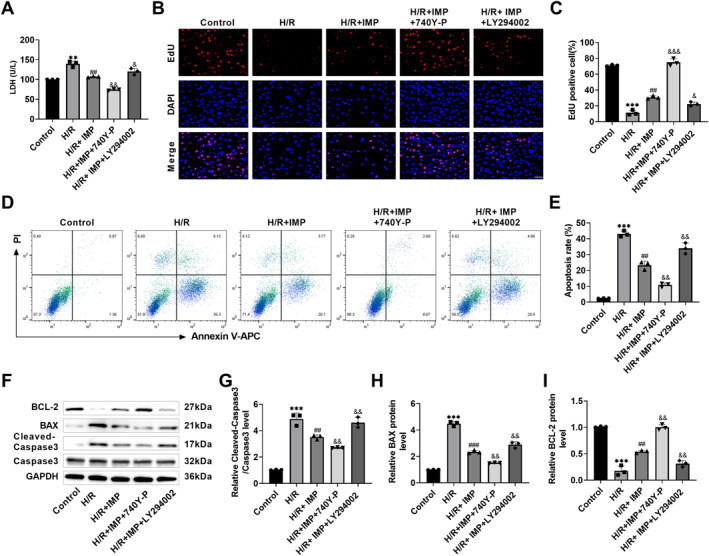
IMP ameliorated H/R‐induced cardiomyocyte apoptosis through the PI3K/AKT/Nrf2 signaling pathway. (A) The H9c2 cytotoxicity was detected by LDH kits. LDH content decreased after 740 Y‐P treatment and increased after LY294002 treatment. (B and C) The cell multiplication was determined through EdU staining. The EdU positive cell number increased after 740 Y‐P treatment and decreased after LY294002 treatment (×40, 50 μm). (D and E) The apoptosis levels were determined by flow cytometry, which decreased after 740 Y‐P treatment and increased after LY294002 treatment. (F–I) The apoptosis‐related protein levels were detected by western blot. After 740 Y‐P treatment, cleaved caspase 3 and BAX expressions lessened, and BCL‐2 expression raised significantly. After LY294002 treatment, the above protein levels were reversed. *n* = 3, **p < 0.01, and ***p < 0.001 versus control group; ##*p* < 0.01 and ###*p* < 0.001 versus H/R group; &*p* < 0.05, &&*p* < 0.01, and &&&*p* < 0.001 versus H/R + IMP group. Due to the limited sample size, statistical comparisons involving more than two groups should be interpreted with caution; the reported *p*‐values were considered exploratory. LDH, lactate dehydrogenase.

In order to illustrate the specific mechanism of the PI3K/AKT/Nrf2 signaling pathway in the cardioprotective effect of IMP, IMP, 740 Y‐P, and LY294002 were used to intervene H9c2 cells for 24 h, and then, H/R was used to induce MIRI. The results showed that compared with IMP intervention, 740 Y‐P treatment showed a trend toward reducing LDH content (Figure 4A) and the H9c2 cell apoptosis rate (Figure 4D, E), and increasing EdU positive cells (Figure 4B, C), indicating that 740 Y‐P could further enhance the effect of IMP on promoting H9c2 cell proliferation and inhibiting apoptosis. However, LY294002 weakened the cardioprotective effect of IMP, LDH content increased (Figure 4A), EdU positive cells decreased (Figure 4B, C), and the apoptosis rate increased (Figure 4D, E). After 740 Y‐P treatment, cleaved caspase 3 and BAX protein expressions declined, and BCL‐2 level elevated. After LY294002 treatment, apoptotic protein levels were reversed (Figure 4F–I). This indicates that IMP reduced cardiomyocyte apoptosis via the PI3K/AKT/Nrf2 signaling.

### 3.5 IMP ameliorated H/R‐induced ERS via the PI3K/AKT/Nrf2 pathway

**FIGURE 5:**
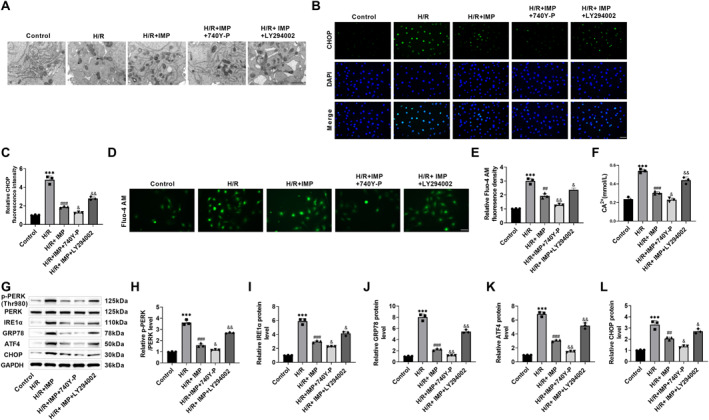
IMP ameliorated H/R‐induced ERS by the PI3K/AKT/Nrf2 signaling. (A) ER morphology was observed by a transmission electron microscope. 740 Y‐P could further improve ER damage, and LY294002 could cause ER swelling and damage (×15.0k, 1 μm). (B and C) The ERS marker CHOP level was assayed through immunofluorescence. The fluorescence intensity of CHOP decreased after 740 Y‐P intervention and increased after LY294002 intervention (×40, 50 μm). (D and E) Intracellular Ca^2+^ concentration was detected by the Fluo‐4 AM fluorescent probe, which decreased after 740 Y‐P intervention and increased after LY294002 intervention (×40, 50 μm). (F) The intracellular Ca^2+^ content was determined with a calcium assay kit. The content of Ca^2+^ decreased after 740 Y‐P intervention and increased after LY294002 intervention. (G–L) Western blot was used to detect the change trend of ERS regulatory proteins. The expression of p‐PERK, IRE1α, glucose‐regulated protein 78, activating transcription factor 4, and CHOP protein decreased after 740 Y‐P intervention and increased after LY294002 intervention. *n* = 3; ***p < 0.001 versus control group; ##*p* < 0.01 and ###*p* < 0.001 versus H/R group; and &*p* < 0.05 and &&*p* < 0.01 versus H/R + imperatorin group. Due to the limited sample size, statistical comparisons involving more than two groups should be interpreted with caution; the reported *p*‐values were considered exploratory. CHOP, C/EBP homologous protein; ER, endoplasmic reticulum; ERS, endoplasmic reticulum stress.

This research also found that compared with IMP intervention, the ER damage in H9c2 cells was further improved after 740 Y‐P intervention, the swelling almost disappeared, and the omentum structure returned to normal; after LY294002 treatment, the ER structure was damaged, the ER was swollen, the ER cavity was dilated, and the omentum structure was destroyed (Figure 5A). The fluorescence intensity of ERS marker CHOP decreased after 740 Y‐P intervention and increased after LY294002 treatment (Figure 5B, C), indicating that IMP may improve H/R‐mediated ERS by the PI3K/AKT/Nrf2 pathway. Secondly, Fluo‐4 AM fluorescence intensity and Ca^2+^ concentration in H9c2 cells decreased after 740 Y‐P intervention and increased after LY294002 treatment (Figure 5D–F). Finally, ERS regulatory proteins p‐PERK, IRE1α, GRP78, ATF4, and CHOP expressions decreased after 740 Y‐P intervention and increased after LY294002 treatment (Figure 5G–L). Therefore, IMP improved H/R‐induced ERS by regulating cytoplasmic Ca^2+^ homeostasis and ERS‐regulated protein expression in cardiomyocytes through the PI3K/AKT/Nrf2 pathway.

### 3.6 IMP improved cardiac injury in MIRI mice through the PI3K/AKT/Nrf2 pathway

**FIGURE 6:**
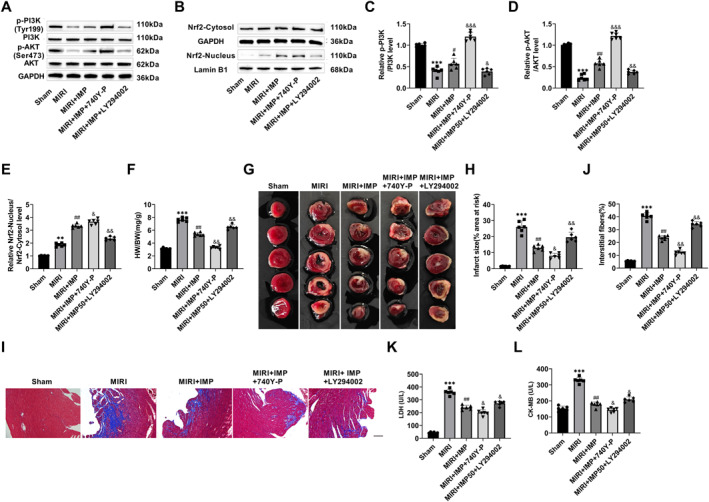
IMP improved cardiac injury in MIRI mice via the PI3K/AKT/Nrf2 pathway. (A–E) The change trend of the PI3K/AKT/Nrf2 pathway protein was detected by western blot. IMP notably raised p‐PI3K, p‐AKT, and Nrf2 nucleus expressions. (F) The mouse heart was quickly stripped and weighed, and the heart weight/body weight was calculated. It was significantly increased in MIRI mice and decreased after IMP treatment. (G and H) Myocardial infarction area in mice was determined by TTC staining. Quantitative analysis of the infarct size expressed as infarct area/area at risk (%). IMP significantly reduced myocardial infarct size in MIRI mice. (I and J) Myocardial fibrosis area in mice was assessed by Masson staining, which was significantly increased in MIRI mice and significantly decreased after IMP treatment (×20, 100 μm). (K and L) Serum CK‐MB and lactate dehydrogenase contents in mice were discovered by kits. They increased significantly in MIRI mice and decreased significantly after IMP intervention. *n* = 6, **p < 0.01, and ***p < 0.001 versus Sham group; #*p* < 0.05 and ##*p* < 0.01 versus MIRI group; and &*p* < 0.05, &&*p* < 0.01, and &&&*p* < 0.001 versus MIRI + IMP group. IMP, imperatorin; MIRI, myocardial ischemia/reperfusion injury.

In this study, the heart protecting influence of IMP was verified again by MIRI mice. The MIRI model was established by coronary artery ligation, and IMP, LY294002, and 740Y‐P were injected intraperitoneally 3 h before MIRI injury. In the myocardium of MIRI mice, p‐PI3K and p‐AKT proteins were significantly lowered, Nrf2 cytosol level was not changed, and Nrf2 nucleus level was notably increased. IMP markedly raised p‐PI3K, p‐AKT, and Nrf2 nucleus expressions. Compared with IMP treatment, p‐PI3K, p‐AKT, and Nrf2 nucleus proteins were significantly increased after 740Y‐P intervention and significantly decreased after LY294002 intervention (Figure 6A–E). After reperfusion injury, the heart weight/body weight (HW/BW) (Figure 6F) and myocardial infarction size (IA/AAR, %) (Figure 6G, H) were significantly increased, and IMP reduced HW/BW and myocardial infarction size. HW/BW and myocardial infarct size were further reduced after 740Y‐P intervention and significantly increased after LY294002 intervention (Figure 6F–H). Myocardial fibers of MIRI mice were significantly thickened and broken, the deposition of blue collagen fibers was notably raised, and the fibrosis area was significantly elevated. With IMP intervention, the myocardial fibers of mice were more complete and clear, and the blue collagen fiber deposition and fibrosis area were significantly reduced. After 740Y‐P intervention, the myocardial fibers of mice were intact and clear, and the fibrosis area was further markedly lessened. After LY294002 intervention, the myocardial fibers of mice were thickened and broken, and the area of fibrosis increased (Figure 6I, J). This indicated that IMP improved myocardial pathological injury of MIRI mice through the PI3K/AKT/Nrf2 pathway. CK‐MB and LDH are markers of myocardial injury. The results showed that serum CK‐MB and LDH levels were significantly increased in MIRI mice and decreased after IMP treatment. Compared with IMP treatment, the contents of CK‐MB and LDH decreased after 740Y‐P intervention and increased significantly after LY294002 intervention (Figure 6K–L). In conclusion, IMP reduced the degree of myocardial fibrosis and improved myocardial injury in MIRI mice by the PI3K/AKT/Nrf2 axis.

### 3.7 IMP reduced myocardial apoptosis and ERS in mice through the PI3K/AKT/Nrf2 signaling pathway

**FIGURE 7:**
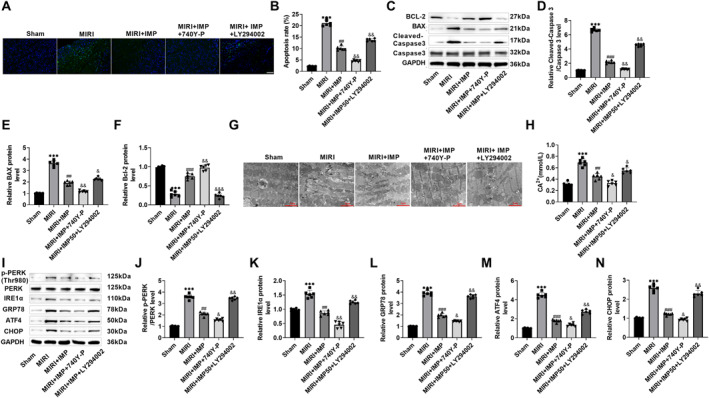
IMP reduced myocardial apoptosis and endoplasmic reticulum stress in mice via the PI3K/AKT/Nrf2 signaling. (A and, B) Cardiomyocyte apoptosis of mice was detected by TUNEL staining, which was raised in MIRI mice and significantly decreased after IMP intervention (×20, 100 μm). (C–F) Western blot detection of apoptosis‐related protein in myocardial tissue of mice. IMP significantly reduced the levels of cleaved caspase 3, and BAX proteins and increased the level of BCL‐2 protein. (G) Transmission electron microscope analysis of mitochondrial morphology in mouse myocardial tissue. Obvious ER expansion and vacuolization were observed in MIRI mice. After IMP treatment, the swelling and vacuolization of ER were alleviated, and the structure of ER was relatively complete (×15.0k, 2 μm). (H) The concentration of Ca^2+^ in myocardial tissue was measured by a calcium determination kit. It increased significantly in MIRI mice and decreased significantly after IMP intervention. (I–N) Western Blot detection of the change trend of ERS regulatory proteins. p‐PERK, IRE1α, glucose‐regulated protein 78, activating transcription factor 4, and C/EBP homologous protein levels were significantly increased in MIRI mice and significantly decreased after IMP intervention. *n* = 6; ***p < 0.001 versus Sham group; ##*p* < 0.01 and ###*p* < 0.001 versus MIRI group; and &*p* < 0.05, &&*p* < 0.01, and &&&*p* < 0.001 versus MIRI + IMP group. ER, endoplasmic reticulum; IMP, imperatorin; MIRI, myocardial ischemia/reperfusion injury.

Many TUNEL‐positive cells were observed in the peri‐infarct area of the MIRI group, suggesting a large number of apoptotic cardiomyocytes. IMP could significantly slow down the apoptosis of cardiomyocytes (Figure 7A, B). Moreover, cleaved caspase 3 and BAX expressions of MIRI mice were markedly elevated, while BCL‐2 level was notably declined. After IMP treatment, apoptotic protein levels were notably reversed, which further indicated that IMP could significantly reduce MIRI‐induced cardiomyocyte apoptosis (Figure 7C–F). Compared with IMP treatment, 740Y‐P intervention further slowed the apoptosis of cardiomyocytes, while LY294002 intervention promoted the apoptosis of cardiomyocytes (Figure 7A–F). TEM results showed that sheet and tubular ER could be observed in normal mice. Obvious ER expansion was observed in MIRI mice, and the ER structure was destroyed and vacuolated, indicating that reperfusion might cause severe ERS response. After IMP treatment, a part of the ER was dilated and swollen, vacuolization was alleviated, and the ER structure was relatively complete. After 740Y‐P treatment, the swelling and vacuolization of ER were almost invisible, and the structure of ER was intact. After LY294002 treatment, the ER showed expansion swelling and vacuolization again (Figure 7G). In addition, the concentration of Ca^2+^ in myocardial tissue increased significantly in MIRI mice and decreased after IMP treatment (Figure 7H); the expression levels of p‐PERK, IRE1α, GRP78, ATF4, and CHOP were obviously raised in MIRI mice and decreased after IMP intervention (Figure 7I–N), indicating that IMP could reduce ERS in MIRI mice. Compared with IMP treatment, Ca^2+^ concentration and the expression levels of‐PERK, IRE1α, GRP78, ATF4, and CHOP decreased again after 740Y‐P treatment and increased after LY294002 treatment (Figure 7H–N). Therefore, IMP reduced myocardial apoptosis and ERS in MIRI mice by the PI3K/AKT/Nrf2 pathway.

## 4 DISCUSSION

Clinically, timely reperfusion therapy is the most effective way for AMI. However, with the blockage of blood vessels or coronary stenosis, it is very easy to cause ischemia‐reperfusion injury, leading to myocardial injury or the expansion of infarct size.^25^, ^26^ The pathogenesis of MIRI is more complicated, which makes it particularly important to further explore the coping strategies of MIRI intervention.^27^ H9c2 cells are derived from embryonic rat hearts and are currently considered to be a good carrier for the establishment of myocardial cell models. Coronary artery ligation is a common method for the establishment of the MIRI mouse model, which has the advantages of good stability and similarity to the pathogenesis of human MIRI. Therefore, in this experiment, H9c2 cells were selected to establish a cardiomyocyte hypoxia model, and a mouse MIRI model was established by LAD artery ligation to identify the protection and the mechanism of IMP on myocardial infarction.

CK‐MB and LDH are mainly present in myocardial cells; only myocardial cell injury or necrosis may be released into the blood in large quantities, so CK‐MB and LDH are important indicators for judging myocardial cell injury. LDH content in H9c2 cells increased significantly after H/R induction, CK‐MB, and LDH contents of MIRI mice increased, and myocardial infarction and myocardial fibrosis area raised obviously. After IMP treatment, the myocardial infarction and fibrosis area decreased, and serum CK‐MB and LDH contents decreased. The results suggested that IMP could reduce myocardial infarction area and protect cardiac function of MIRI.

ER exists in the cytoplasm and is an important place for protein synthesis, folding, modification, and storage of Ca^2+^. Maintaining ER homeostasis is important for cell survival and normal physiological functions. When the body is stimulated by external signals such as hypoxia, calcium overload, and energy metabolism disorders, it can cause an imbalance in ER homeostasis, causing a large accumulation of unfolded and misfolded proteins in the ER to occur in ERS. The ER quality control system will initiate the unfolded protein response (UPR) to identify misfolded proteins and initiate proteases to degrade them. However, when ERS is persistent and severe, general regulation has been unable to rebuild the steady state, and UPR will induce cells that produce a large number of misfolded proteins to enter the apoptotic process.^28^ Studies have shown that myocardial ischemia‐reperfusion can cause ERS, and ERS‐induced apoptosis is a key mechanism of myocardial dysfunction caused by MIRI. Studies have shown that inhibition of ERS can inhibit cardiomyocyte apoptosis and protect cardiomyocytes.^29^ Therefore, inhibiting ERS‐induced cardiomyocyte apoptosis is an effective strategy to reduce MIRI.

GRP78 is a molecular chaperone in the ER and participates in the UPR in ERS. When the unfolded protein in the ER accumulates, GRP78, which should be combined with PERK, ATF6, etc., binds to the unfolded protein and activates the ERS signaling pathway.^30^ PERK is present in the ER membrane; it is phosphorylated and activated after dissociation from GRP78; it inhibits mRNA transcription and protein synthesis and increases the expression of CHOP protein that regulates apoptosis by increasing transcription factor 4 transcription.^31^ ATF6 and GRP78 are activated in the Golgi apparatus and migrate to the nucleus after dissociation, which can upregulate the ER chaperone gene.^32^ Caspase 12/Caspase 3 and CHOP signaling pathways are ERS‐specific apoptosis pathways. Caspase 12 is activated only when ERS occurs, and the increase of its content can indicate the initiation of the ER apoptosis program. After activation, caspase 3 can be cleaved to activate caspase 3. Cleaved caspase 3, as an executor with pro‐apoptotic ability, can reflect the degree of apoptosis.^33^ CHOP is a marker protein of ERS apoptosis and the most important regulator of ER pro‐apoptotic function. Continuous and intense ERS can simultaneously activate three UPR pathways to promote CHOP gene transcription. CHOP can induce the apoptosis cascade by inhibiting anti‐apoptotic protein BCL‐2 and regulating the homodimerization of pro‐apoptotic protein BAX. It is inferred that IMP may inhibit cardiomyocyte apoptosis by decreasing ER‐related proteins and regulating ERS. Liang et al. showed that MIRI was improved by inhibiting the expression of ATF4, CHOP, and apoptotic proteins, reducing apoptosis and ERS.^32^ The results of this research showed that in H/R‐induced H9c2 cells and myocardial tissue of MIRI mice, the ER was dilated and swollen; the ER structure was destroyed; vacuolization occurred; Ca^2+^ concentration was significantly increased; p‐PERK, IRE1α, GRP78, ATF4, CHOP, cleaved caspase 3, and BAX expressions were also elevated; BCL‐2 level was markedly reduced; and the myocardial cell apoptosis rate was significantly increased. IMP treatment dose‐dependently reduced the swelling and vacuolation of ER, restored the structural integrity of ER, down‐regulated the concentration of intracellular Ca^2+^ , and suppressed the expression of ER stress‐related and apoptotic proteins, thereby inhibiting cardiomyocyte apoptosis. More importantly, H/R specifically activated caspase 12, an initial caspase that is only cleaved and activated under ERS conditions and is not involved in death receptors or mitochondrial apoptosis pathways. This suggests that ERS may mediate apoptosis. In short, IMP can suppress ERS and its mediated apoptosis in cardiomyocytes, thereby improving MIRI.

The PI3K/AKT is the primary pathway in MIRI, which resists oxidative stress, apoptosis, inflammation, and autophagy through various pathways.^34^ Studies have shown that in the MIRI mouse model, activating the PI3K/AKT pathway and upregulating p‐PI3K and p‐AKT expressions can reduce cardiac inflammation and cardiomyocyte apoptosis.^35^, ^36^ According to the literature, the PI3K/AKT pathway can be activated by stimulation such as myocardial injury and stress. When myocardial ischemia occurs, the upstream gene PI3K of this pathway is first activated under extracellular signal stimulation. The activated PI3K phosphorylates the Thr308 and Ser473 sites of AKT to activate AKT.^37^ Phosphorylated AKT is an important mediator in this signaling pathway, which can phosphorylate a series of substrates to activate the downstream complex.^38^ Nrf2 is usually used as a downstream effector molecule of the PI3K/AKT signaling. AKT can act as an upstream target of Nrf2 to regulate Nrf2, so PI3K indirectly regulates Nrf2. As an important transcription factor, Nrf2 can regulate cells to respond to various harmful reactions; induce the expression of endogenous protective factors such as antioxidant protein genes, antioxidant defense factors, and anti‐inflammatory factors; and is vital for cardiac protection.^39^ Nrf2 is a key target for protecting MIRI.^40^ Nrf2‐KO mice exhibit deteriorated infarct size and cardiac function after ischemia‐reperfusion, possibly due to a lack of the antioxidant system.^41^ Another study found that the Nrf2/HO‐1 pathway promotes nuclear translocation and phosphorylation of Nrf2, promotes AMPK phosphorylation, and reduces apoptosis.^42^ This underscores the importance of Nrf2 in alleviating MIRI. Similar to the results of Zhao et al.,^43^ in this experiment, p‐PI3K and p‐AKT protein expressions were obviously lowered in H/R‐induced H9c2 cells and myocardial tissue of MIRI mice; Nrf2 cytosol level was not changed; and Nrf2 nucleus protein level and NQO1 and HO‐1 RNA expression levels were slightly raised. However, p‐PI3K, p‐AKT, and Nrf2 nucleus protein expressions and NQO1 and HO‐1 RNA expression levels were markedly increased with IMP treatment, indicating that IMP significantly promoted p‐PI3K and p‐AKT proteins and the nuclear translocation of Nrf2. Moreover, after using the PI3K/AKT agonist 740 Y‐P, cardiomyocyte apoptosis and ERS were inhibited, the ER damage was further improved, the expansion swelling and vacuolization were almost invisible, and the ER structure was intact. However, after the use of the PI3K/AKT inhibitor LY294002, the ER showed expansion, swelling, and vacuolization again, and ERS and cardiomyocyte apoptosis increased significantly. This indicates that IMP may reduce myocardial apoptosis and ERS through the PI3K/AKT/Nrf2 pathway, thereby improving MIRI and exerting myocardial protection.

In addition to the PI3K/AKT pathway, the MAPK family (including ERK1/2, JNK, and p38) also plays critical roles in MIRI, with cross talk between MAPK and ER stress signaling pathways.^44^ While the PI3K/AKT pathway is robustly protective in rodents, its efficacy and role as a primary protective mediator have not been consistently recapitulated in studies involving larger mammals or in clinical settings, highlighting important species‐specific differences in cardioprotective signaling.^45^, ^46^ Therefore, while our results delineate a plausible mechanism for IMP's action in preclinical models, they underscore the necessity for future validation in more translationally relevant systems to assess the potential applicability of targeting this pathway for human MIRI therapy.

Several limitations of this study should be acknowledged. Due to the significant pathophysiological differences between genders in ischemic heart disease, this study was only conducted in male mice, and subsequent experiments will verify this conclusion in female mice with different physiological states (such as normal estrous cycle and ovariectomized model). Second, our mechanistic exploration was primarily focused on ERS‐mediated apoptosis via the PI3K/AKT/Nrf2 axis. While this pathway is significant, MIRI involves a complex interplay of multiple processes, including but not limited to other forms of regulated cell death (e.g., necroptosis and ferroptosis), inflammation, and mitochondrial dysfunction. Whether IMP exerts protective effects through these parallel or alternative mechanisms was not addressed in the current study. Third, the sample size for in vitro experiments (*n* = 3 per group) is smaller for multigroup comparisons, which may compromise statistical power. Although each biological replicate comprised 3 technical replicates, the degrees of freedom for group comparisons remain limited. Consequently, the statistical significance observed in cell‐based assays should be interpreted as exploratory rather than confirmatory. Future studies with larger sample sizes (*n* ≥ 6 per group) are warranted to validate these findings. In addition, in order to enhance clinical significance, the effects of IMP on ischemic postconditioning (performed at the beginning of reperfusion) and delayed treatment (6 h after myocardial infarction) models will be studied in the future.

## 5 CONCLUSION

In summary, IMP can reduce MIRI myocardial damage by the PI3K/AKT/Nrf2 pathway, and its mechanism may be useful to protect cardiomyocytes by attenuating ERS‐induced apoptosis. This study offers novel ideas for explaining the pathogenesis of MIRI and further verifies the therapeutic effect of IMP on myocardial cells of MIRI. In addition, the PI3K/AKT/Nrf2 pathway is also hoped to become a novel strategy for clinically treating MIRI and a new idea for drug development.

## AUTHOR CONTRIBUTION

**Mingjun Han**: Developed and planned the study, performed experiments, and interpreted results. Edited and refined the manuscript with a focus on critical intellectual contributions. **Fan Yu**: Participated in collecting, assessing, and interpreting the date. Made significant contributions to date interpretation and manuscript preparation. **Huanhuan Li**: Provided substantial intellectual input during the drafting and revision of the manuscript.

## CONFLICT OF INTEREST STATEMENT

The authors declare no conflicts of interest.

## ETHICS STATEMENT

This study was approved by the Hospital of Chengdu University of Traditional Chinese Medicine Ethic Committee (Grant No. KY‐‐202401032).

## CONSENT TO PUBLISH

The manuscript has neither been previously published nor is under consideration by any other journal. The authors have all approved the content of the paper.

## Data Availability

All data generated or analyzed during this study are included in this article. Further inquiries can be directed to the corresponding author.
